# Regulation of HCN2 Current by PI3K/Akt Signaling

**DOI:** 10.3389/fphys.2020.587040

**Published:** 2020-11-09

**Authors:** Zhongju Lu, Hong Zhan Wang, Chris R. Gordon, Lisa M. Ballou, Richard Z. Lin, Ira S. Cohen

**Affiliations:** ^1^Department of Physiology and Biophysics, Stony Brook University, Stony Brook, NY, United States; ^2^Department of Medicine, Stony Brook University, Stony Brook, NY, United States; ^3^Department of Nephrology, Stony Brook University, Stony Brook, NY, United States; ^4^Medical Service, Northport VA Medical Center, Northport, NY, United States

**Keywords:** HCN2, pacemaker current, PI3K, Akt, sinus node

## Abstract

It has long been known that heart rate is regulated by the autonomic nervous system. Recently, we demonstrated that the pacemaker current, I_*f*_, is regulated by phosphoinositide 3-kinase (PI3K) signaling independently of the autonomic nervous system. Inhibition of PI3K in sinus node (SN) myocytes shifts the activation of I_*f*_ by almost 16 mV in the negative direction. I_*f*_ in the SN is predominantly mediated by two members of the HCN gene family, HCN4 and HCN1. Purkinje fibers also possess I_*f*_ and are an important secondary pacemaker in the heart. In contrast to the SN, they express HCN2 and HCN4, while ventricular myocytes, which do not normally pace, express HCN2 alone. In the current work, we investigated PI3K regulation of HCN2 expressed in HEK293 cells. Treatment with the PI3K inhibitor PI-103 caused a negative shift in the activation voltage and a dramatic reduction in the magnitude of the HCN2 current. Similar changes were also seen in cells treated with an inhibitor of the protein kinase Akt, a downstream effector of PI3K. The effects of PI-103 were reversed by perfusion of cells with phosphatidylinositol 3,4,5-trisphosphate (the second messenger produced by PI3K) or active Akt protein. We identified serine 861 in mouse HCN2 as a putative Akt phosphorylation site. Mutation of S861 to alanine mimicked the effects of Akt inhibition on voltage dependence and current magnitude. In addition, the Akt inhibitor had no effect on the mutant channel. These results suggest that Akt phosphorylation of mHCN2 S861 accounts for virtually all of the observed actions of PI3K signaling on the HCN2 current. Unexpectedly, Akt inhibition had no effect on I_*f*_ in SN myocytes. This result raises the possibility that diverse PI3K signaling pathways differentially regulate HCN-induced currents in different tissues, depending on the isoforms expressed.

## Introduction

The sinoatrial node (SN) initiates electrical activity in the heart that triggers the cardiac contractions responsible for circulation of the blood. This electrical activity includes action potentials associated with contraction and a period between action potentials called diastolic depolarization. The SN rate is dependent on both voltage-dependent ion channels (called the membrane voltage clock) and triggered release of intracellular Ca^2+^ (called the Ca^2+^ clock) (DiFrancesco, 1993; Lakatta et al., 2010). All of the elements necessary for this electrical event are intrinsic to the SN myocytes.

The membrane voltage clock includes a number of ion channels which generate net inward current during the diastolic interval, driving the SN toward the action potential threshold. One of these diastolic membrane currents is the pacemaker current, I_*f*_. I_*f*_ is selective to Na^+^ and K^+^ and activates slowly on hyperpolarization (DiFrancesco, 1993). The cardiac pacemaker channels are comprised of heteromultimers of three members of the HCN gene family. HCN4 is the dominant isoform in rabbit SN myocytes (81% of the total HCN mRNA), while HCN1 (18%) and HCN2 (0.6%) are present at lower levels (Shi et al., 1999). By contrast, HCN2 is the predominant isoform in ventricular myocytes, and I_*f*_ in Purkinje fibers is mediated by HCN2 and HCN4 (Shi et al., 1999; Han et al., 2002).

An increase in I_*f*_ increases the SN pacing rate. The sympathetic nervous system increases heart rate in part by shifting the activation of I_*f*_ to more positive potentials, allowing greater activation during diastolic depolarization. The parasympathetic nervous system has the opposite effect on both heart rate and the voltage dependence of I_*f*_ (DiFrancesco, 1993). Although the details of this rapid sympathetic and parasympathetic regulation of heart rate and I_*f*_ have been studied in detail, little is known about other regulatory mechanisms that control intrinsic heart rate over longer time periods of time. One recent study pointed to a potential role for AMPK in this process (Yavari et al., 2017). Our own studies indicated that phosphoinositide 3-kinase (PI3K) regulates both SN rate and I_*f*_ independently of the autonomic nervous system (Lin et al., 2019).

Class I PI3Ks produce the lipid second messenger phosphatidylinositol 3,4,5-trisphosphate [PI(3,4,5)P_3_]. The enzymes are heterodimers containing one of four distinct catalytic subunits bound to one of several regulatory subunits. PI3Ks in the Class IA subgroup can be activated by tyrosine kinases (TKs) *via* binding of the regulatory subunits to tyrosine-phosphorylated proteins. There are multiple downstream effectors of PI3K, including the protein kinase Akt (Ballou et al., 2015). We recently demonstrated that inhibition of PI3K by the pan PI3K isoform inhibitor PI-103 induced a slowing of sinus rate *in situ* and *in vitro* and a negative shift in activation of I_*f*_ in SN myocytes. There was no change in the whole cell conductance of I_*f*_ (Lin et al., 2019). This work built on a previous study which demonstrated that some TK inhibitors (TKIs) act through inhibition of PI3K signaling to induce an acquired long QT syndrome (Lu et al., 2012). PI3K inhibition caused alterations in a number of cardiac membrane currents, including a reduction in amplitude of I_*Na*_, I_*Kr*_, and I_*Ks*_ together with a reduction in I_*CaL*_ and a positive shift in its activation. An increase in the persistent sodium current I_*NaP*_ was also observed (Lu et al., 2012). Earlier work showed that TKIs also caused a simple reduction in amplitude of I_*f*_ in SN myocytes (Wu and Cohen, 1997). However, the consequences of TK inhibition on heterologously expressed HCN isoforms varied widely, with no effect on HCN1, reduced amplitude and slowed activation kinetics of both HCN2 and HCN4, and a shift in the activation of HCN2 to more negative potentials (Yu et al., 2004). These differences in the effects of TK inhibition on different HCN isoforms, plus the fact that one of the downstream actions of TK inhibition can be a reduction in PI3K activity, induced us to study PI3K regulation of heterologously expressed HCN2, the dominant ventricular isoform (Shi et al., 1999).

## Materials and Methods

### Electrophysiology

#### Patch Clamp Recording of HCN2 Current Expressed in HEK293 Cells

**FIGURE 1:**
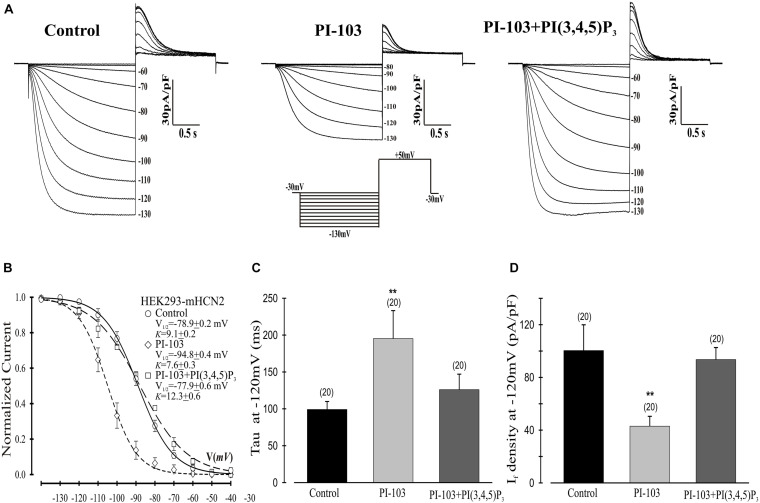
Effects of PI-103 and PI(3,4,5)P_3_ on I_*f*_ in HEK293 cells stably expressing mHCN2. **(A)** Traces of I_*f*_ activation in control cells or cells incubated for 2 h with 1 μM PI-103 or treated with PI-103 plus infusion of 1 μM PI(3,4,5)P_3_ in the patch pipette. Inset: Voltage clamp protocol for activating I_*f*_ (see text for details). **(B)** Boltzmann fit of 1-s isochronal activation curves for I_*f*_ shows that PI-103 negatively shifted the voltage at half-maximal activation and infusion of PI(3,4,5)P_3_ reversed the shifted midpoint to near the control level. **(C)** The mean time constants (tau) from a single exponential fit of I_*f*_ measured at –120 mV. **(D)** Summary data show a reduction of I_*f*_ amplitude by PI-103 which was reversed by PI(3,4,5)P_3_. I_*f*_ was measured at –120 mV from a holding potential of –30 mV. Numbers above the bars show the number of cells examined. **, *P* < 0.05.

**FIGURE 2:**
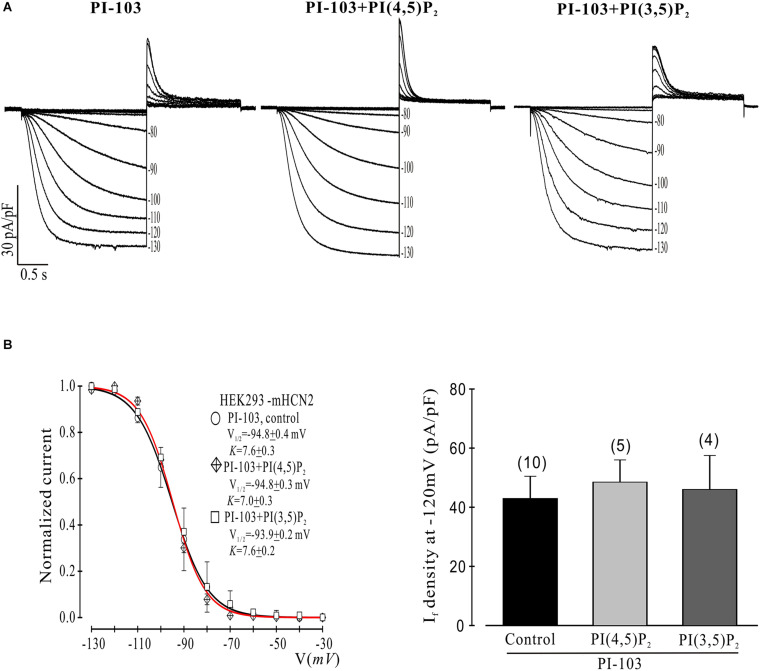
Control phosphoinositides do not reverse PI-103 inhibition of mHCN2 current in HEK293 cells. **(A)** Original traces of PI-103-induced inhibition of mHCN2 current activation. Cells were incubated for 2 h at 37°C with 1 μM PI-103, with or without additional infusion of control phospholipid (1 μM) in the patch pipette solution. The voltage protocol for current activation is described in the text. **(B)** Activation curves from summarized data (left panel). The red curve is the fit to the PI-103 + PI(3,5)P_2_ data. Summarized data show I_*f*_ density in the presence of PI-103 was not increased by control phospholipids (right panel). Numbers above the bars indicate the number of cells examined.

**FIGURE 3:**
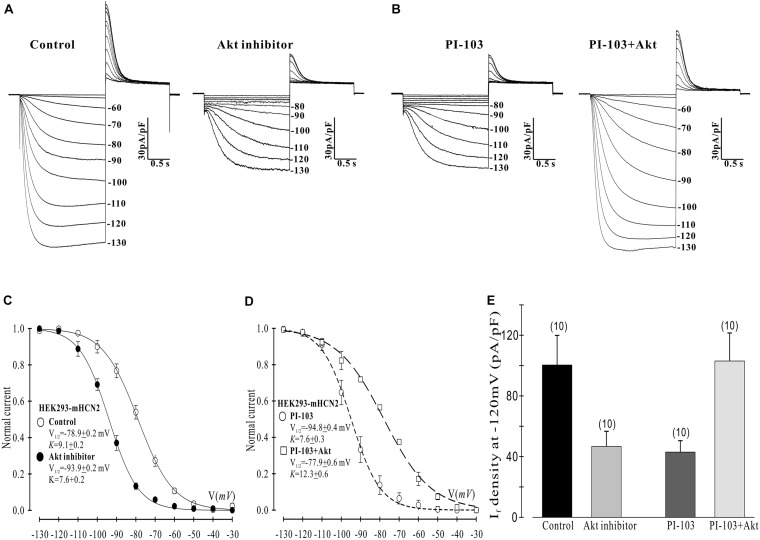
Akt rescues the PI-103-induced inhibition of mHCN2 current in HEK293 cells. **(A,B)** Activation of mHCN2 currents. Cells were incubated for 2 h at 37°C with 10 μM Akt inhibitor (Akti) or 1 μM PI-103, or treated with PI-103 plus infusion of 20 nM active Akt1. The voltage protocol for current activation is described in the text. **(C,D)** Activation curves from summarized data. **(E)** Summarized data show a reduction of I_*f*_ density by the Akt inhibitor and rescue of the PI-103-induced reduction of mHCN2 current by Akt1. Numbers above the bars indicate the number of cells examined.

**FIGURE 4:**
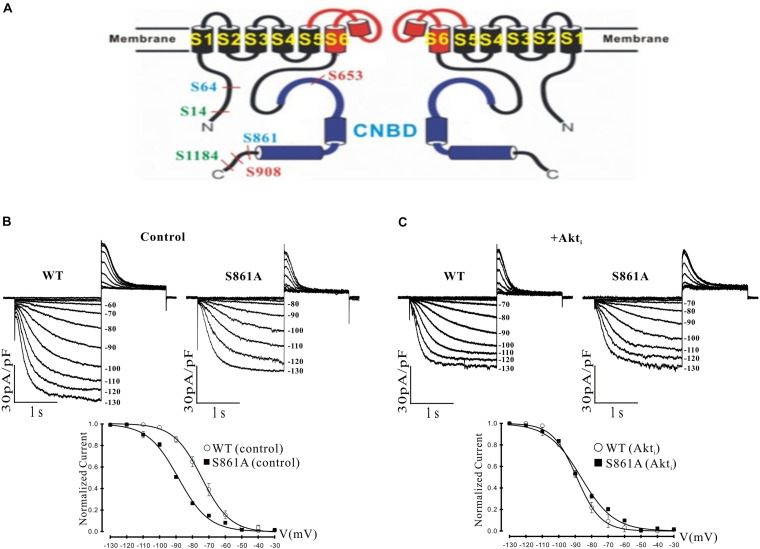
Effect of a putative Akt phosphorylation site on mHCN2-induced current. **(A)** Potential Akt phosphorylation sites in HCN proteins. Shown are two of the four subunits that form HCN channels and the position of serines in HCN1 (red), HCN2 (blue), and HCN4 (green) located within consensus sequences for Akt phosphorylation. The pore and S6 transmembrane segments are red and the cyclic nucleotide-binding domain (CNBD) is blue. **(B,C)** Mutation of serine 861 to alanine (S861A) in mHCN2 causes an inhibition of expressed current in HEK293 cells. **(B)** Upper panels, sample traces of activation of transiently expressed WT and mutant mHCN2. Note a more negative activation threshold (–80 mV in S861A vs. –60 mV for WT) and a reduced current density at voltages (–130 mV) with maximal current activation. Lower panel, activation curves for WT and S861A mHCN2 fit with the Boltzmann equation. The *V*_1__/__2_ values were -74.2 ± 0.4 mV (*n* = 10 cells, WT) and –88.6 ± 0.1 mV (*n* = 7 cells, S861A). The corresponding slope factors were 8.6 ± 0.4 mV and 9.2 ± 0.1 mV, respectively. The voltage protocol is described in the text. **(C)** Upper panels, sample traces of activation of transiently expressed WT and mutant mHCN2 in the presence of Akti (10 μM). Lower panel, activation curves for WT and S861A mHCN2 fit with the Boltzmann equation. Cells were incubated for 2 h at 37°C with Akti. The *V*_1__/__2_ values were -89.1 ± 0.3 mV (*n* = 7 cells, WT with Akti) and –86.5 ± 0.7 mV (*n* = 7 cells, S861A with Akti). The corresponding slope factors were 7.4 ± 0.3 mV and 10.0 ± 0.7 mV, respectively. Note that Akti caused an inhibition of expressed WT mHCN2 and a negative shift in voltage dependence but no further inhibition of the expressed mutant current.

We used a traditional whole-cell patch clamp (voltage-clamp) recording technique to examine HCN2 currents expressed in HEK293 cells. Experiments in Figures 1–3 employed a stable cell line expressing mouse HCN2 and GFP. The experiments in Figure 4 used HEK293 cells transiently transfected with GFP-mHCN2 constructs (see Section 2.2). Whole-cell patch clamp recording was performed using the Axopatch-1D amplifier coupled to the pCLAMP data acquisition and analysis software package (version 10, Axon Instruments, Inc). Patch electrode resistance was 4-6 MΩ and room temperature was 20 ± 1°C. The bath solution was (in mM): NaCl 140, KCl 5.4, MgCl_2_ 1, CaCl_2_ 1.8, HEPES 10, and glucose 10 (pH 7.4 with NaOH). The pipette solution contained: aspartate 130, KOH 146, NaCl 10, EGTA-KOH 5, CaCl_2_ 2, Mg-ATP 2, and HEPES-KOH 10 (pH 7.2 with KOH) (Potapova et al., 2004). The voltage protocol to measure the HCN2 activation curve was to hold the cell at -30 mV and hyperpolarize for 1.5 s to voltages between −30 and −130 mV in 10-mV increments, followed by a 1-s voltage step to + 50 mV to record the tail currents, followed by a step back to −30 mV. The normalized plot of tail current versus test voltage was fit with a Boltzmann function, and then, the voltage at half maximum activation (*V*_1__/__2_) and slope factor (*K*) were calculated from the fit. Current densities are expressed as the value of the time-dependent component of current amplitude measured at the end of the test potential (-120 mV, 1.5-s duration) from a holding potential of -30 mV and normalized to cell membrane capacitance.

#### Whole-Cell Patch Clamp Recording of I_*f*_ in Single SN Myocytes

Single SN myocytes were isolated from rabbit hearts as previously described (Lin et al., 2019). All animal-related procedures were approved by the Stony Brook University Institutional Animal Care and Use Committee. Electrophysiological measurements of I_*f*_ were performed at room temperature (20 ± 1°C). The external solution contained (in mM): NaCl 140, KCl 8, CaCl_2_ 1.8, MgCl_2_ 1.0, HEPES 5, glucose 10, MnCl_2_ 2, CdCl_2_ 0.2, and BaCl_2_ 8 (pH adjusted to 7.4 with NaOH). The internal solution contained KCl 50, K-aspartate 80, MgCl_2_ 1, EGTA 10, Mg-ATP 3, and HEPES 10 (pH 7.2). Mn^2+^ and Cd^2+^ were used to reduce Ca^2+^ currents, which can overlap with and obscure I_*f*_ tail currents. Ba^2+^ was used to block the background K^+^ current (I_*K*__1_), which activates and inactivates in the same voltage range as I_*f*_. The liquid junction potential (10 mV) between the electrode tip and cell interior was not corrected. The voltage-clamp protocol for I_*f*_ activation was to apply 1-s hyperpolarizing voltage steps ranging from -30 mV to -110 mV in -10-mV increments from a holding potential of -30 mV and then to apply a depolarizing step to + 50 mV for 0.5 s to record the tail currents, after which the preparation was stepped back to the holding potential. To generate the I_*f*_ activation curve, the normalized plot of tail current versus test voltage was fit with a Boltzmann function, and *V*_1__/__2_ and *K* were calculated from the fit.

#### Cell Treatments

PI(3,4,5)P_3_ or control phosphoinositides were diluted in internal solution to a final concentration of 1 μM and infused through the patch pipette. Where indicated, cells were pretreated with PI-103 (1 μM) or Akti (10 μM) for 2 h before patch clamping. HEK293 cells were treated with PI-103 or Akti at 37°C, and isolated SN cells were treated with Akti at room temperature.

#### Statistical Analysis

When comparing different sets of data, statistical analysis was performed with either Student’s t tests or ANOVA; significance was set to P ≤ 0.05. Results are given as mean ± SEM.

### DNA Constructs

Mouse *Hcn2* cDNA was subcloned into pEGFP-C1, and the putative mHCN2 Akt phosphorylation site mutant, S861A, was made by PCR. The 50 μl PCR reaction contained 1x Ultra AD buffer (Stratagene), 200 μM of each dNTP, 30 ng DNA template, 0.25 μM of each primer, 1.5 μl PfuUltra AD DNA polymerase (Stratagene), and 3% dimethylsulfoxide. The PCR program was: (1) 95°C for 1 min; (2) 95°C for 1 min; (3) 68°C for 1 min; (4) 72°C for 9 min; (5) 13x to step 2; (6) 72°C for 10 min; (7) hold at 4°C. The mutation was confirmed by sequencing both DNA strands. The primers (Eurofins Genomics) used for mutagenesis were 5′-CTCGCGCCTCTCTGCCAACTTGTGACC (forward) and 5′-GGTCACAAGTTGGCAGAGAGGCGCGAG (reverse). The constitutively active myristoylated Akt construct (Ballou et al., 2001) was subcloned into pcDNA3.1(−).

### Western Blotting and Immunoprecipitation

HEK293 cells were transiently transfected with the appropriate constructs using *Trans*IT-239 transfection reagent (Mirus Bio) and analyzed 2-3 days later. For western blotting, the cell layers were rinsed twice with PBS on ice and then scraped into a lysis buffer containing 50 mM HEPES, pH 7.5, 10 mM sodium pyrophosphate, 50 mM NaCl, 50 mM NaF, 5 mM EDTA, 0.1 mM sodium orthovanadate, 0.25% sodium deoxycholate, 1% IGEPAL CA-630, 200 μM phenylmethylsulfonyl fluoride, 1.7 μg/ml aprotinin and 2 μg/ml leupeptin. After strong vortexing, the lysates were centrifuged for 10 min at 4°C at 18,000 × g. Protein assays (Bio-Rad) were done on the supernatants, and equal amounts of protein were either directly subjected to SDS-PAGE or subjected to immunoprecipitation using GFP antibody. For immunoprecipitation, lysate supernatant was mixed with antibody and left on ice overnight, then protein G-agarose beads (Roche) were added. After rotating at 4°C for 2 h, the beads were washed 4 times with lysis buffer, boiled with SDS sample buffer, and subjected to SDS-PAGE. The proteins were transferred onto PVDF by semi-dry blotting, and signals were detected on film using horseradish peroxidase-linked secondary antibodies (GE Healthcare) and Western Lightning Plus ECL reagent (PerkinElmer). After signal detection, the blot was stripped at 50°C for 30 min in 62.5 mM Tris, 2% SDS, and 100 mM 2-mercaptoethanol, pH 6.7, reblocked in 5% non-fat dry milk in 20 mM Tris, 137 mM NaCl, and 0.1% Tween 20, pH 7.6, and probed with another antibody.

### Reagents and Antibodies

Chemical agents employed in the studies include the pan-PI3K inhibitor PI-103 (Cayman Chemical), Akt inhibitor VIII (Akti; Millipore), and the diC8 phosphoinositides PI(3,4,5)P_3_, phosphatidylinositol 3,5-bisphosphate (PI(3,5)P_2_) and phosphatidylinositol 4,5-bisphosphate (PI(4,5)P_2_) from Echelon Biosciences. The antibodies used targeted GFP (Santa Cruz sc-9996), phospho-Ser/Thr Akt substrate sites (Cell Signaling 9614), Akt phospho-Thr308 (Cell Signaling 9275), or HCN2 (Abcam ab19346). Active recombinant Akt1 protein was from Millipore.

## Results

We began by investigating the effects of PI-103, which inhibits all four isoforms of class I PI3K, on mHCN2-induced currents. As indicated in Figure 1, exposure of cells expressing mHCN2 to PI-103 reduced the amplitude of the mHCN2-induced current (panel A) and shifted the voltage dependence of activation in the negative direction (panel B). The voltage at half-maximal activation (*V*_1__/__2_) was −78.9 ± 0.2 mV (control) versus −94.8 ± 0.4 mV (PI-103), a difference of 15.9 mV. These actions were prevented when PI(3,4,5)P_3_, the second messenger produced by PI3K, was present in the pipette solution (Figure 1). To confirm that this was a specific effect of the PI3K second messenger, we performed additional experiments with control phosphoinositides PI(3,5)P_2_ or PI(4,5)P_2_ in the pipette solution. The results are provided in Figure 2. Panel A shows the raw data. Neither PI(3,5)P_2_ nor PI(4,5)P_2_ prevented the actions of PI-103. Panel B shows an absence of effect of either control phospholipid on the voltage dependence of activation, as well as an absence of effect on the current amplitude at −120 mV, where activation is nearly complete and the current activates rapidly enough to reach steady state during the test pulse.

Next we examined which downstream elements in the PI3K signaling pathway might mediate the decrease in mHCN2-induced conductance and the shift in the voltage dependence of activation to more negative potentials. We began by examining the actions of an Akt inhibitor (Akti) on mHCN2-induced current. The results are provided in Figure 3. Panel A provides raw data which indicate that Akti produced virtually identical effects on the mHCN2-induced currents as PI-103. This result suggested that Akt acts downstream of PI3K to regulate mHCN2 channels. In support of this hypothesis, pipette perfusion of active Akt protein in cells pretreated with PI-103 completely reversed the inhibitory effects of PI-103 (panel B). Panel C illustrates that Akti caused a 15 mV negative shift in *V*_1__/__2_ as compared to the control, while panel D demonstrates that active Akt reversed the shift caused by PI-103. Finally, the effects of Akti alone or active Akt in the presence of PI-103 on mHCN2-induced current amplitude is provided in panel E. These results confirm that Akt functions downstream of PI3K to regulate the mHCN2-induced current.

It was possible that the actions of Akt were exerted through direct phosphorylation of the mHCN2 channel. We identified a number of potential Akt phosphorylation sites in HCN proteins, as illustrated in panel A of Figure 4. Interestingly, the sequence surrounding S861 at the C terminus of mHCN2 (RSRLSS_861_NL) is identical in human, dog and zebrafish, and similar highly conserved sequences are found at the C termini of HCN1 (KPRFAS_908_NL in mHCN1) and HCN4 (RSKLPS_1199_NL in mHCN4). To determine if Akt regulates mHCN2 by acting on S861, we made a non-phosphorylateable alanine mutation at this site. The S861A mHCN2 mutant recapitulated both the reduction in amplitude and negative shift in activation voltage dependence of the mHCN2-induced current caused by treatment of the wild-type channel with PI-103 or Akti (panel B). This result suggested that the mutation prevents Akt-mediated phosphorylation and regulation of the channel. Indeed, Akti had no effect on the S861A mHCN2 current (panel C). As indicated in panel A, S861 is located at the C terminus, close to the cAMP binding site.

**FIGURE 5:**
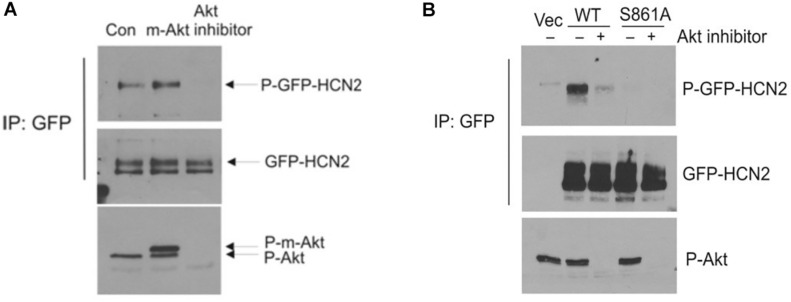
Akt-mediated phosphorylation of GFP-mHCN2 protein. **(A)** HEK293 cells stably expressing GFP-HCN2 were transfected with vector (Con) or myristoylated Akt (m-Akt) and analyzed 2 days later or treated with 10 μM Akt inhibitor for 30 min. Cell lysates were subjected to immunoprecipitation with GFP antibody and the immunoprecipitated proteins were blotted for phospho-Ser/Thr Akt substrate sites (top) and HCN2 (middle). Cell lysates were analyzed with a phospho-Thr308 Akt antibody to assess the activation state of the enzyme (bottom). m-Akt migrates just above endogenous Akt. **(B)** Akt-mediated phosphorylation of GFP-HCN2 is abolished in the S861A mutant. HEK293 cells were transiently transfected with empty GFP vector (Vec), WT GFP-HCN2 or S861A GFP-HCN2. Two days later, the cells were treated with vehicle or 10 μM Akti for 1 h. Cell lysates were subjected to immunoprecipitation with GFP antibody, and the immunoprecipitated proteins were blotted for phospho-Ser/Thr Akt substrate sites (top) and GFP-HCN2 using HCN2 antibody (middle). The P-GFP-HCN2 signal comigrated with the upper band on the GFP-HCN2 blot. Cell lysates were analyzed with phospho-Thr308 Akt antibody to assess the activation state of the enzyme (bottom).

We next attempted to confirm the importance of this phosphorylation site by western blotting. We immunoprecipitated GFP-mHCN2 from cell lysates and then probed western blots of the immunoprecipitated proteins with an antibody that recognizes phosphorylated Akt consensus sites (RXXS-P/T-P). Panel A of Figure 5 (upper blot) shows that expression of a constitutively active form of Akt (m-Akt) in cells stably expressing GFP-mHCN2 slightly increased the phosphorylation of the channel protein at these sites, whereas treatment with Akti abolished the signal. The middle blot demonstrates that similar amounts of GFP-mHCN2 protein were loaded in each lane, while the bottom blot demonstrates that phosphorylation (i.e., activation) of Akt and m-Akt was high in cells under control conditions, but nearly undetectable in cells treated with Akti. Panel B compares the response to Akti treatment of transiently transfected WT *vs.* S861A GFP-mHCN2. The upper blot shows that immunoprecipitates of cells transfected with empty vector contained a minimal level of protein containing phosphorylated Akt substrate sites, as expected. The strong signal for WT GFP-mHCN2 under control conditions was greatly reduced by Akti treatment, whereas S861A GFP-HCN2 did not appear to contain phosphorylated Akt substrate sites under either condition. The middle blot shows that the amount of channel protein was equivalent in the WT and S861A lanes, and the bottom blot shows that Akt was equally active in the WT and S861A cells and that Akti eliminated the activity of Akt. These results suggest that S861 may be the major Akt phosphorylation site in mHCN2. An alternative explanation is that mutation of S861 negatively affects Akt phosphorylation of another site.

**FIGURE 6:**
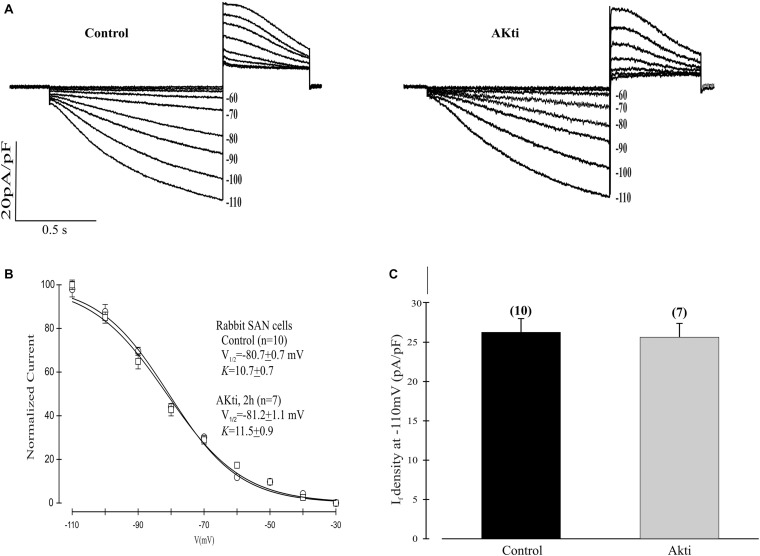
Effect of Akti on I_*f*_ in SN cells isolated from rabbit heart. **(A)** Traces of I_*f*_ activation in control cells (left panel) or cells incubated for 2 h with 10 μM Akti at room temperature (right panel). **(B)** Boltzmann fit of 1-s isochronal activation curves for I_*f*_ shows that Akti did not shift the voltage at half-maximal activation. The *V*_1__/__2_ values were -80.7 ± 0.7 mV (*n* = 10 cells, control) and –81.2 ± 1.1 mV (*n* = 7 cells, Akti). The corresponding slope factors were 10.7 ± 0.7 mV and 11.5 ± 0.9 mV, respectively. **(C)** Summary data show Akti did not affect I_*f*_ amplitude. I_*f*_ was measured at –110 mV from a holding potential of –30 mV.

Given the dominant effect of PI3K/Akt signaling on the mHCN2 isoform, we next decided to investigate whether Akti exerts similar effects on I_*f*_ in rabbit SN myocytes. The results are provided in Figure 6. The raw currents in panel A indicate little or no effect of the inhibitor on I_*f*_. This observation was confirmed by the absence of a shift in the voltage dependence of activation (panel B) or in the amplitude of the expressed current at −110 mV (panel C).

## Discussion

Although there is a clear understanding of the mechanism by which SN rate responds to changes in demand *via* the autonomic nervous system, much less is known about the determinants of basal heart rate. Basal heart rate is important, as it decreases with age, and this decrease can result in simple bradycardia as well as sick sinus syndrome (Adán and Crown, 2003). Recently, it was reported that the protein kinase AMPK may play a role in regulating basal heart rate by affecting I_*f*_ and Ca^2+^ release in the SN (Yavari et al., 2017). We reported that PI3K signaling also affects basal heart rate (Lin et al., 2019). PI3K inhibition slowed the SN rate both *in situ* and *in vitro* and shifted the activation of I_*f*_ to more negative potentials. By contrast, artificially increasing PI3K signaling by addition of PI(3,4,5)P_3_ induced a positive shift in I_*f*_. These shifts in voltage dependence were independent of and larger than those caused by autonomic agonists (Lin et al., 2019). A negative shift in the voltage dependence of I_*f*_ as well as a reduction in I_*f*_ magnitude have also been seen in mice with aging (Larson et al., 2013).

Proteins in the HCN family assemble as homotetramers or heterotetramers to form functional channels. We showed that heterologously expressed homotetramers of HCN1, HCN2 or HCN4 are differentially regulated by TKIs. The TKI genistein had no effect on HCN1, reduced the current magnitude of HCN4, and reduced the magnitude and caused a negative shift in the activation of HCN2 (Yu et al., 2004). Because TKs can activate PI3K signaling, we decided to investigate the effects of a PI3K inhibitor on HCN2 channels. Unlike HCN4 and HCN1, which are prominent in the SN, HCN2 plays a dominant role in ventricular myocytes. Similar to the results with genistein, we found that PI-103 reduced the amplitude and negatively shifted the activation of mHCN2 current. These effects were reversed by infusing PI(3,4,5)P_3_ in the pipette, but not PI(3,5)P_2_ or PI(4,5)_2_. Selective reversal by PI(3,4,5)P_3_ strongly suggests that the action of PI-103 is due to inhibition of PI3K. The positive effect of PI(4,5)P_2_ on HCN2 currents seen under some conditions (Pian et al., 2006) thus apparently cannot compensate for the loss of Akt-induced phosphorylation of HCN2 that we believe underlies the effect of PI-103.

Several pieces of evidence indicate that Akt acts downstream of PI3K to regulate HCN2. First, Akti and PI-103 had nearly identical effects on HCN2, reducing current magnitude and shifting its activation to more negative potentials. Second, an active form of Akt reversed the actions of PI-103. Third, mutation of a putative Akt phosphorylation site in mHCN2 to alanine mimicked the effects of PI-103 or Akti on current amplitude and voltage dependence. The mHCN2 S861A current was also insensitive to Akti. Finally, WT mHCN2 expressed in cells exhibited increased phosphorylation of Akt sites when coexpressed with an activated form of the enzyme and decreased phosphorylation in the presence of Akti. By contrast, mHCN2 S861A did not seem to contain phosphorylated Akt sites.

Given the similar effects of PI-103 on mHCN2 and I_*f*_ in SN myocytes (Lin et al., 2019), we decided to investigate whether Akt also regulates I_*f*_. We were surprised to discover that there was no observable effect of Akti on rabbit SN I_*f*_. One possible explanation for this result is that even though HCN4 and HCN1 in the SN contain possible Akt phosphorylation sites at their C termini as discussed above, these sites do not contribute to channel regulation by PI3K. Alternatively, a kinase distinct from Akt might be responsible for phosphorylating these sites in the SN. SGK1 is one likely candidate, as it is activated downstream of PI3K, phosphorylates the same optimal peptide sequence as Akt, and is insensitive to Akti (Logie et al., 2007; Di Cristofano, 2017). Further studies using heterologous expression systems and SN myocytes are needed to elucidate how PI3K regulates HCN4, HCN1 and I_*f*_.

## Conclusion

In conclusion, our experiments suggest that PI3K regulates homotetrameric mHCN2 channels through Akt-dependent phosphorylation of S861. A reduction in PI3K/Akt activity causes a negative shift in the voltage dependence of activation and a decrease in current magnitude. By contrast, PI3K regulation the HCN4/HCN1 heterotetrameric channels that mediate I_*f*_ in the SN seems to be mediated by an alternative signaling pathway that does not involve Akt. Thus, consistent with previous results reporting differential effects of TKIs on HCN isoforms (Yu et al., 2004), our results suggest that regulation of HCN channels by PI3K/Akt signaling is also isoform specific.

## Data Availability Statement

The raw data supporting the conclusions of this article will be made available by the authors, without undue reservation. Requests to access the datasets should be directed to IC.

## Ethics Statement

The animal study was reviewed and approved by the IACUC Stony Brook University.

## Author Contributions

ZL and HW designed, performed, and analyzed the patch clamp experiments. CG designed, performed, and analyzed the biochemical experiments. LB made constructs, designed, performed, and analyzed the biochemical experiments, and co-wrote the manuscript. RL conceived of the project, analyzed the data, and co-wrote the manuscript. IC conceived the project, analyzed the data, oversaw the execution of the patch clamp experiments, and co-wrote the manuscript. All authors contributed to the article and approved the submitted version.

## Conflict of Interest

The authors declare that the research was conducted in the absence of any commercial or financial relationships that could be construed as a potential conflict of interest.
